# Anatomical characterization of pulmonary artery and implications to pulmonary artery pressure monitor implantation

**DOI:** 10.1038/s41598-023-47612-9

**Published:** 2023-11-22

**Authors:** Hamza Zafar, Dharshan Neelam-Naganathan, Jennifer T. Middleton, Sarah K. Binmahfooz, Christian Battersby, Dominic Rogers, Andrew J. Swift, Alexander M. K. Rothman

**Affiliations:** 1 University of Sheffield, Sheffield, UK; 2 Sheffield University Teaching Hospitals NHS Trust, Sheffield, UK; 3 Division of Clinical Medicine, School of Medicine and Population Health, Beech Hill Road, Sheffield, S10 2RX UK

**Keywords:** Cardiology, Tomography

## Abstract

In patients with heart failure, guideline directed medical therapy improves outcomes and requires close patient monitoring. Pulmonary artery pressure monitors permit remote assessment of cardiopulmonary haemodynamics and facilitate early intervention that has been shown to decrease heart failure hospitalization. Pressure sensors implanted in the pulmonary vasculature are stabilized through passive or active interaction with the anatomy and communicate with an external reader to relay invasively measured pressure by radiofrequency. A body mass index  > 35 kg/m^2^ and chest circumference > 165 cm prevent use due to poor communication. Pulmonary vasculature anatomy is variable between patients and the pulmonary artery size, angulation of vessels and depth of sensor location from the chest wall in heart failure patients who may be candidates for pressure sensors remains largely unexamined. The present study analyses the size, angulation, and depth of the pulmonary artery at the position of implantation of two pulmonary artery pressure sensors: the CardioMEMS sensor typically implanted in the left pulmonary artery and the Cordella sensor implanted in the right pulmonary artery. Thirty-four computed tomography pulmonary angiograms from patients with heart failure were analysed using the MIMICS software. Distance from the bifurcation of the pulmonary artery to the implant site was shorter for the right pulmonary artery (4.55 ± 0.64 cm vs. 7.4 ± 1.3 cm) and vessel diameter at the implant site was larger (17.15 ± 2.87 mm vs. 11.83 ± 2.30 mm). Link distance (length of the communication path between sensor and reader) was shorter for the left pulmonary artery (9.40 ± 1.43 mm vs. 12.54 ± 1.37 mm). Therefore, the detailed analysis of pulmonary arterial anatomy using computed tomography pulmonary angiograms may alter the choice of implant location to reduce the risk of sensor migration and improve readability by minimizing sensor-to-reader link distance.

## Introduction

**Figure 1:**
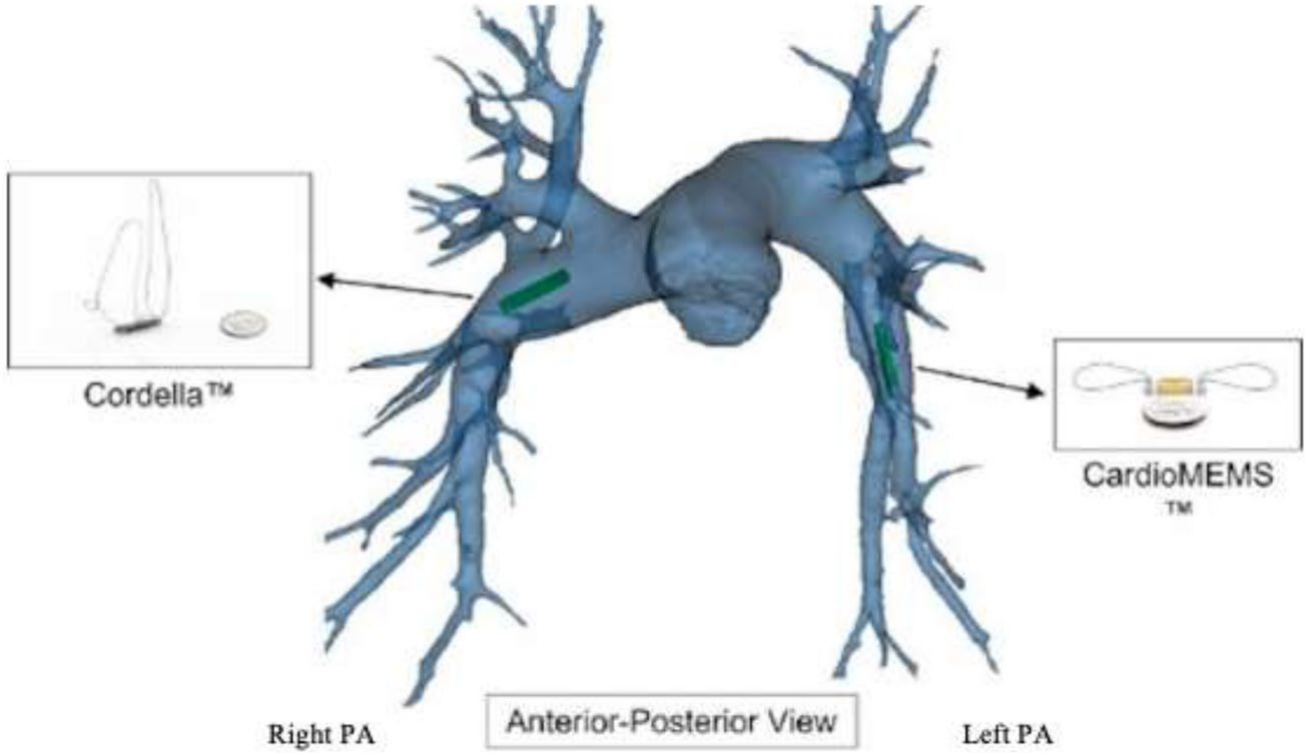
Anatomical location of Cordella Sensor (RPA) and CardioMEMS sensor (LPA) as shown from the Antero-posterior view.

Congestive heart failure (CHF) is a chronic condition associated with significant symptoms and frequent episodes of decompensation that lead to frequent healthcare interactions, hospitalization, and premature death^1,2^. Prognosis remains poor, despite the development of effective pharmacological and non‐pharmacological interventions^3–8^. Increased left ventricular filling pressures precede symptoms of decompensation, making accurate measurements a priority in clinical management^9–11^. Historically, repeated measurements of cardiopulmonary haemodynamics via invasive right heart catheterization (RHC) have been used to guide therapeutic escalation, transition to advanced therapies and to determine eligibility, and inform the timing of, heart transplantation^12^. However, repeated measurements are limited due to the requirement for an invasive, hospital-based procedure. The development of implanted pressure monitoring devices offers the unique opportunity to monitor clinical relavant physiology in patients with CHF, allowing for more timely interventions, and gauging the effect of the interventions in real-time while the patient is in the community. Pulmonary artery pressure monitors are implanted by passing a Swan-Ganz catheter into the pulmonary artery from percutaneous access of the venous system in the jugular or femoral location. Following fluoroscopic angiography of the target area, a guidewire was placed through the access catheter and the delivery catheter advanced to the implantation site in either the left (CardioMEMS) or right (Cordella) pulmonary artery (Fig. 1).

The accuracy and reliability of the pulmonary artery pressure (PAP) readings depend critically on the position of the sensor in the pulmonary vasculature and its relationship to the interrogating device on the anterior chest wall or back. Pressure sensors implanted in the pulmonary vasculature are stabilized through passive or active interaction with the anatomy and communicate with an external reader to wirelessly transmit the invasively measured hemodynamic data using radiofrequency^13^. Pulmonary vasculature anatomy is variable between patients and the size, angulation of vessels, and depth of sensor location from the chest wall is largely unexamined. Sensor implant stability is crucial for accurate readings. However, sensor migration has been observed both at the time of, and after, implantation. Possible mechanisms include placement in a larger than recommended pulmonary artery (PA), a more transverse artery course (rather than sagittal), or implantation in the proximal or distal to the target area within the vessel^14,15^.

The present study describes the anatomy and morphology of the PA in a population of patients with heart failure eligible for pressure monitor implantation to highlight key differences in anatomy relevant to PAP monitor implantation, orientation, link distance and clinical use.

## Methods

### Study population

**Figure 2:**
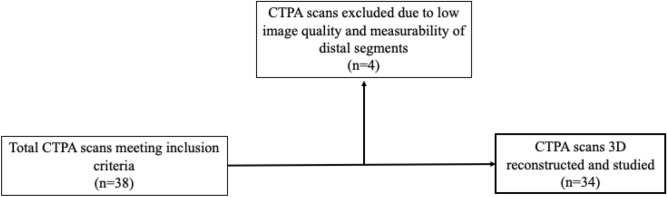
Flowchart describing the scans included in the study and the reasons for the exclusion of the remaining.

A retrospective study of thirty-eight computed tomography pulmonary angiogram (CTPA) scans undertaken at Sheffield Teaching Hospitals NHS Foundation Trust between 2018 and 2019 were analysed to evaluate the dimensions and metrics of the PA. The experimental setup was approved and conducted in accordance with the ASPIRE (Assessing the Severity of Pulmonary Hypertension In a Pulmonary Hypertension Referral Centre) code of ethics and with the institutional review board/ethics committee, REC:16/YH/0352 and the public access to the database is closed. All the methods were carried out in accordance with the relevant guidelines and regulations. The eligible patients were men or women over 18 years old with a diagnosis of New York Heart Association (NYHA) class I-IV heart failure (HF) ≥ 6 months with reduced or preserved ejection fraction at the time of screening. Patients were receiving appropriate medical management for HF for 3 months prior to screening and were clinically stable for at least 1 month prior to study entry. Patients with unrepaired significant congenital heart disease, pulmonary arterial hypertension, pulmonary embolism, deep vein thrombosis, myocardial infarction, stroke, glomerular filtration rate (GFR) < 25 ml/min, renal dialysis, planned for heart transplant or left ventricular assist device or with mechanical right heart valves were excluded from the study. From this, 34 computed tomography (CT) scans were chosen for PA characterization analysis based on the quality of images and measurability at distal segments (Fig. 2).

### CT pulmonary angiogram methods

CT pulmonary angiogram scans were acquired on light-speed 64-slice GE Healthcare multidetector computed tomography (MDCT) scanners and reported by a consultant radiologist prior to reconstruction. The parameters of the imaging included 100 mA with automated dose reduction, 120 kV, pitch 1, with a rotation time of 0.5 s and 0.625 collimation. The field of view used was 400 mm × 400 mm with an acquisition matrix of 512 × 512. For the imaging, 100 ml of Ultravist and Bayer IV contrast agents were used and administered at 5 mL/s. High-resolution CT scans (HRCTs) were reconstructed using contrast-enhanced acquisitions with 1.25 mm collimation from the apex of the lung to the diaphragm^16^.

### Analysis methods

A three-dimensional (3D) model of the PA was constructed through MIMICS medical imaging software using imported patient DICOM images and volume-based 3-D reconstruction was undertaken by a qualified clinician with specific training in the process. A “mask” was created in MIMICS to highlight the area of interest in the images. The “mask” was then split to help differentiate PA from extraneous highlighted segments.

The PA measurements were in made using the MIMICS software. A 3-D model of the PA was constructed and an automated centreline of the vessels identifed. MIMICS is programmed to determine the diameter at any point on the centreline and the segment length and angle at between selected points. By utilizing this program, all measurements were calculated automatically by MIMICS at our preferred points of interest.

The PA metrics evaluated in this study were vessel diameter for both RPA and left pulmonary artery (LPA), the distance of the segment length from the zone where the implantable sensor would be placed in the vessels to the main PA bifurcation, the distance between the sensor and the sensor reader, called the link distance, the angle of the RPA downturn and the chest circumference of each patient. As the 3-D reconstruction of the PA is key for anatomical characterization prior to PAP monitor implantation, a detailed stepwise methodology and quality checks are further described in the online Supplementary Document (“Supplementary information”).

**Figure 3:**
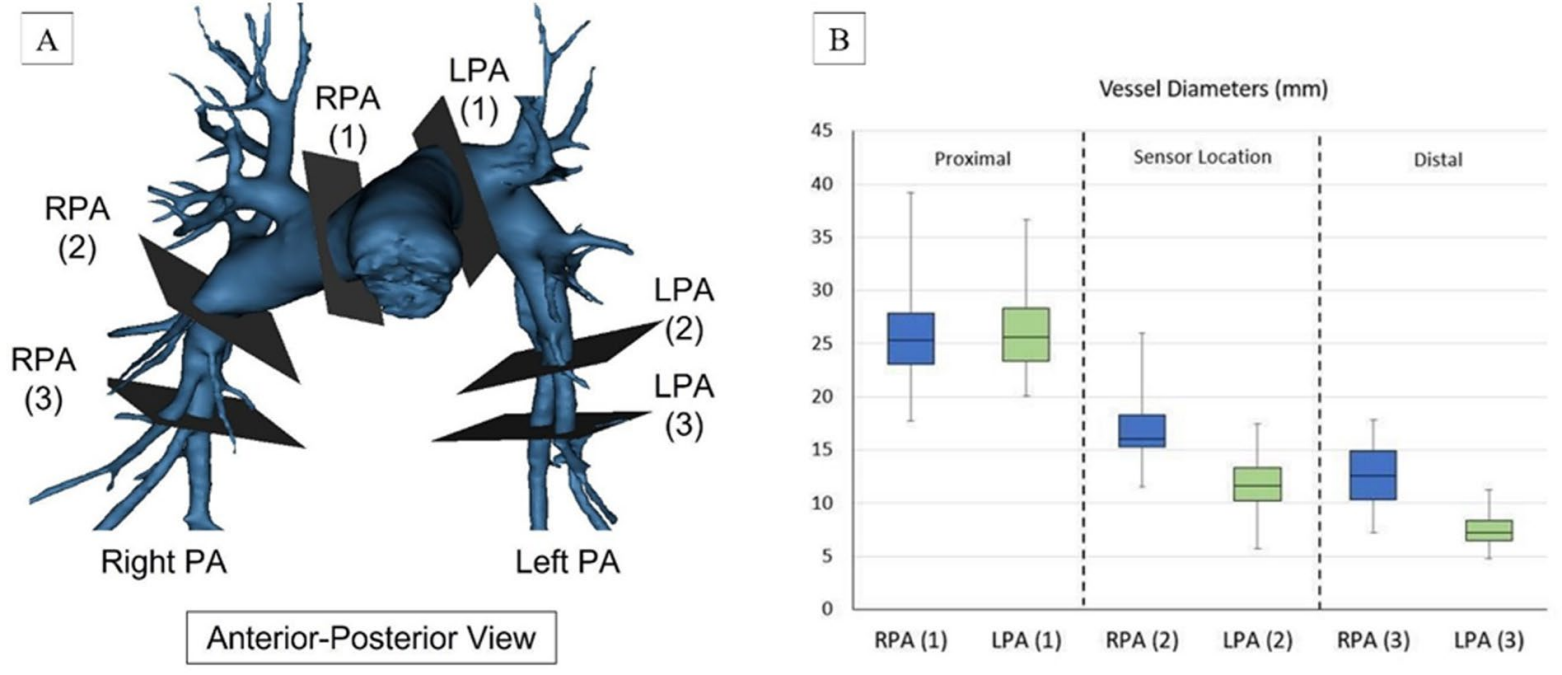
(**A**) PA anatomy zones for analysis viewed from the Antero-posterior view. Zone 1 (Proximal), Zone 2 (Sensor site) and Zone 3 (Distal), (**B**) Comparison of the vessel diameters at each zone for both RPA and LPA. (1) = Proximal, (2) = Sensor site. (3) = Distal.

**Figure 4:**
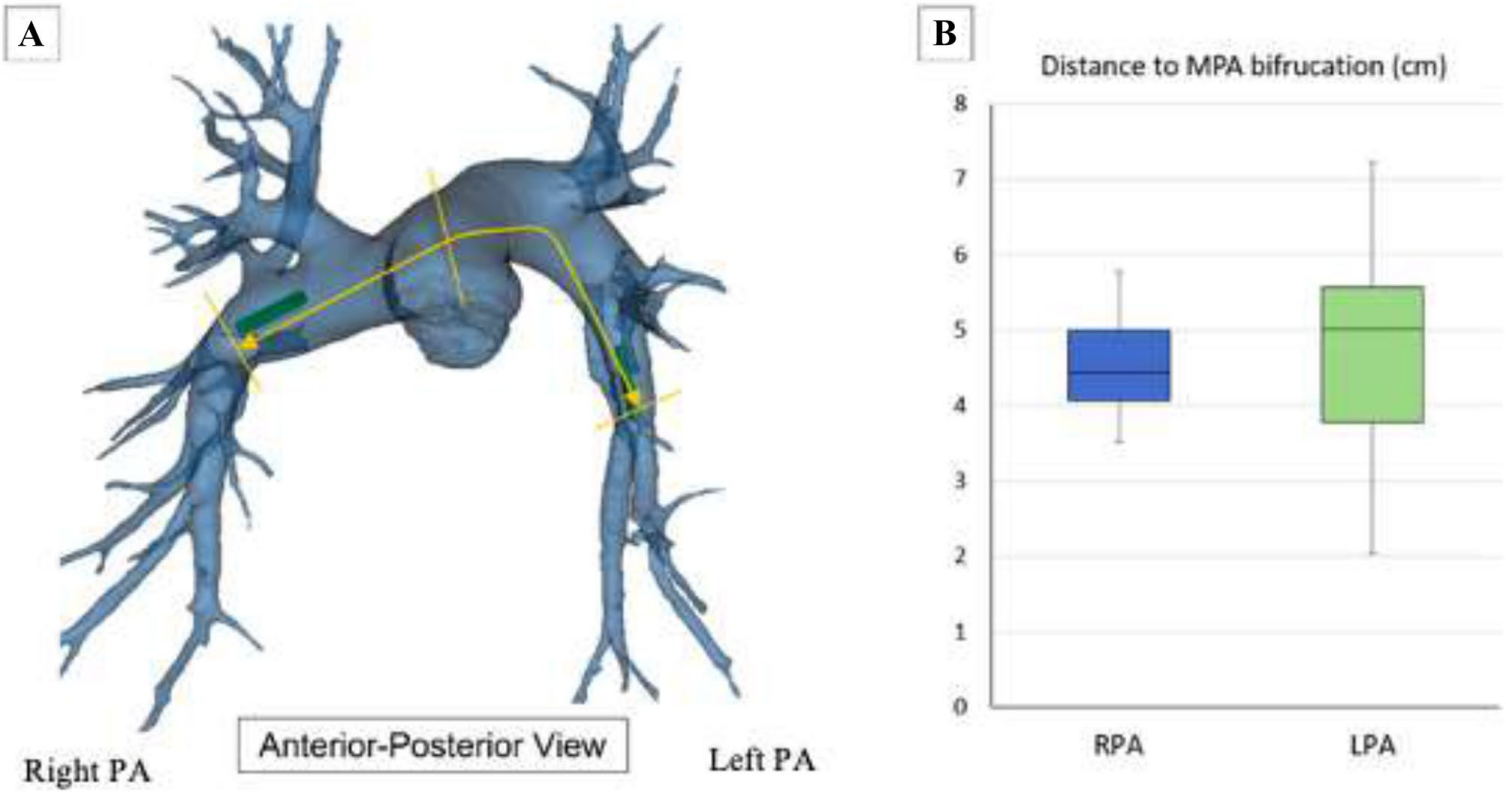
(**A**) Path from main PA bifurcation to sensor location for both RPA (Cordella) and LPA (CardioMEMS) used to measure distance, (**B**) Comparison of distance in centimetres from the main PA bifurcation to each sensor location.

**Figure 5:**
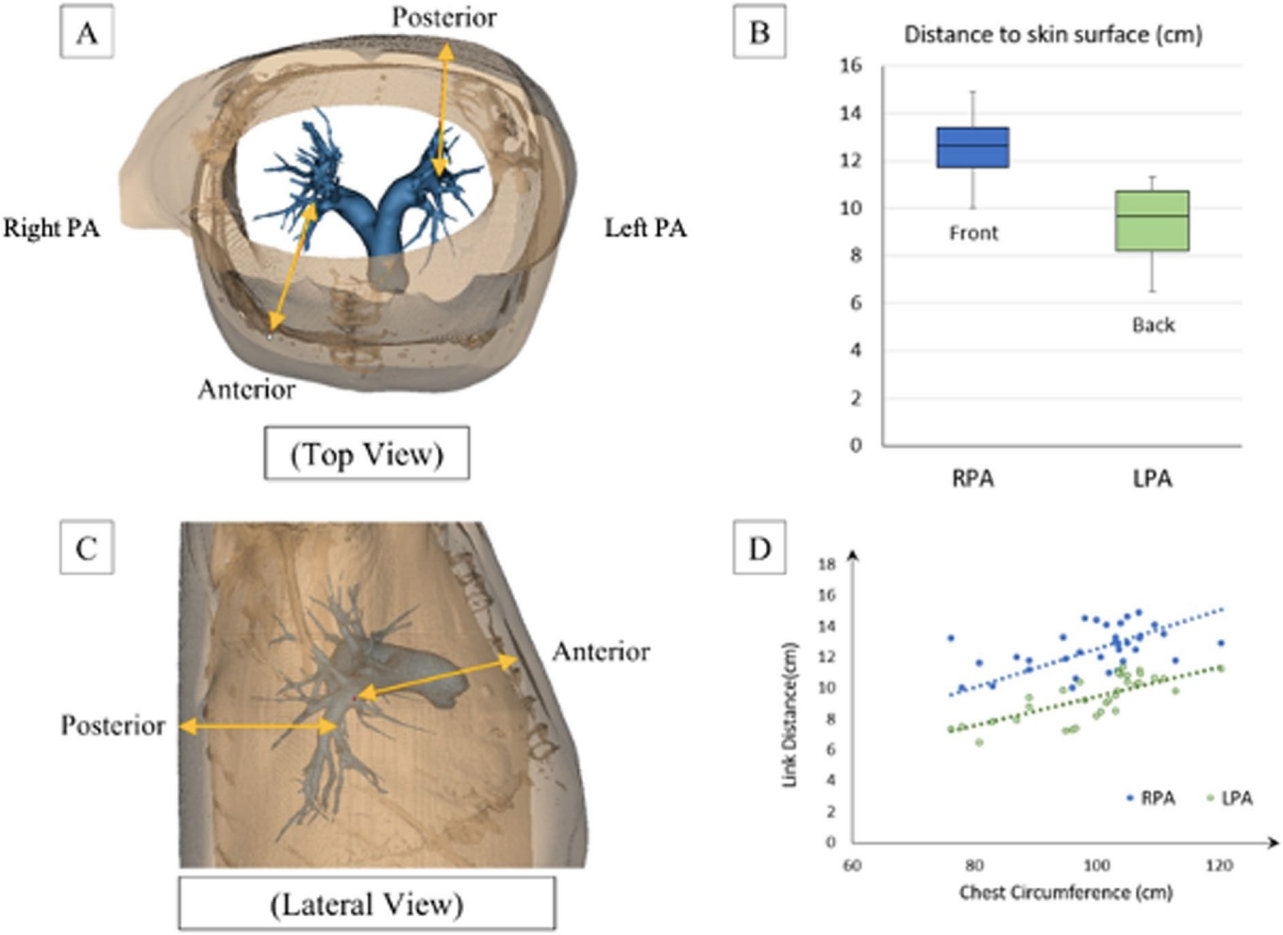
(**A**) Link distance (LD) for Cordella measured from RPA to the anterior chest wall and CardioMEMS measured from LPA to posterior back shown in top view, (**B**) Comparison of LD in centimetres for Cordella (RPA) and CardioMEMS (LPA), (**C**) LD for Cordella measured from RPA to anterior chest wall and CardioMEMS measured from LPA to posterior back shown in lateral view, (**D**) LD for Cordella (RPA) and CardioMEMS (LPA) plotted against chest circumference to assess for correlation.

**Figure 6:**
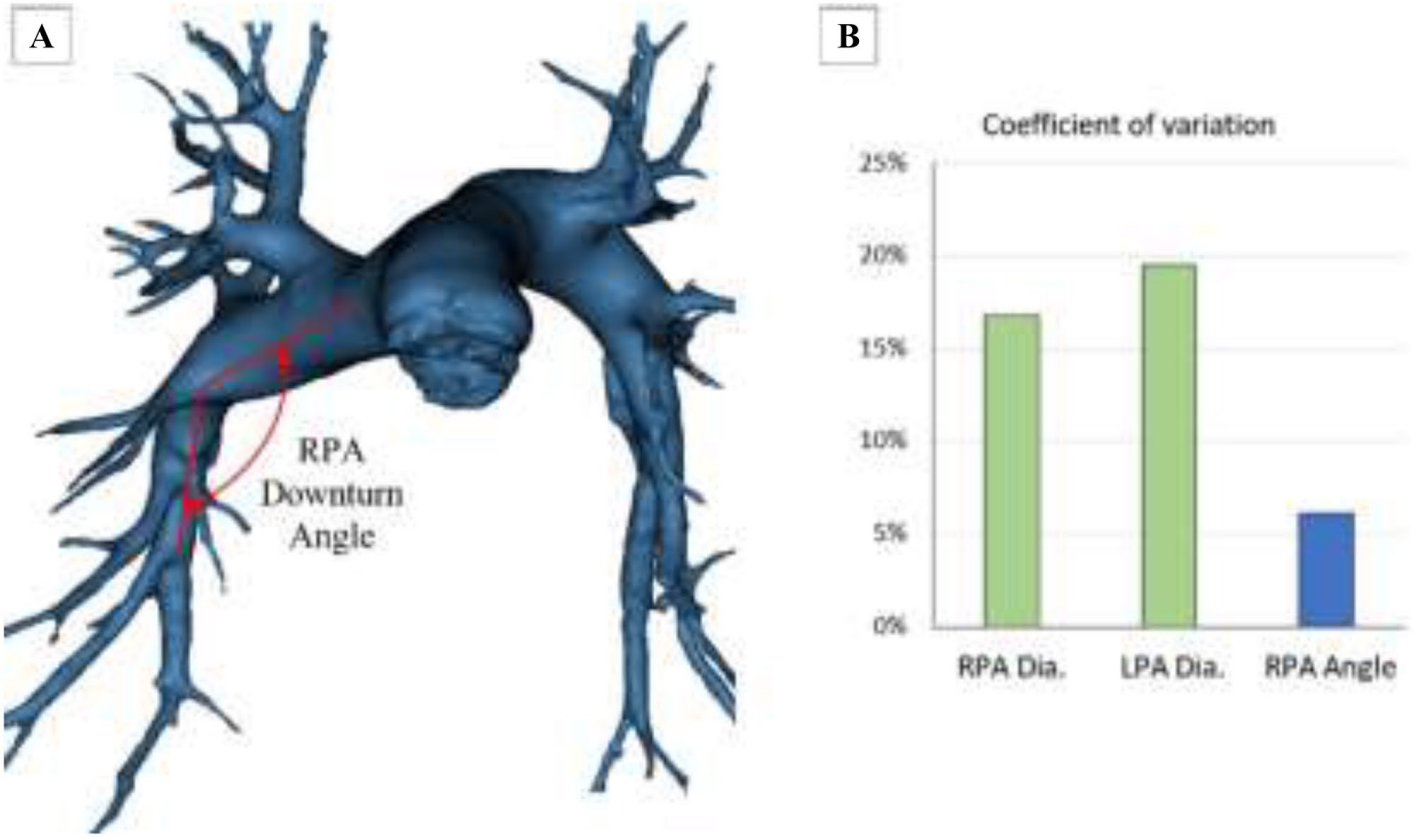
(**A**) RPA downturn angle at the site Cordella sensor implantation, (**B**) Comparison of the coefficient of variation of the RPA diameter, LPA diameter and RPA downturn angle.

The LPA and RPA were divided into 3 zones based on the anatomical landmarks and the diameter of each zone was determined for analysis. Zone 1 (Proximal) was defined as the section on the RPA and LPA between the main PA bifurcation and the first branch of the RPA and LPA and proximal to the sensor deployment zone. Zone 2 (Sensor) was defined as the section distal to Zone 1 i.e., Bifurcation of PA and then the Truncus arteriosus (TA) branch of RPA where the Cordella sensor (RPA) and CardioMEMS (LPA) are deployed. Zone 3 (Distal) was defined to be 2 cm distal to Zone 2 (Fig. 3A). The diameter of each zone in the RPA was calculated as the average of the horizontal and vertical diameters of each zone which were measured along the axial axis in the sagittal view and the coronal axis in the sagittal view, respectively. The diameter of each zone in the LPA was measured diagonally in between the sagittal and coronal sections in the axial view. For the LPA, only one diameter measurement was constructed instead of the average of two different diameters from two different planes respectively. The segment length from the main PA bifurcation to Zone 2 of each vessel was measured along the coronal axis in axial view (Fig. 4A). The chest circumference was also measured in axial view at the PA level using a spline that went along the outer chest wall. The length of the spline was recorded as chest circumference. The link distance (LD) was recorded as the distance from the implantable sensor in each vessel to the skin surface (where a reader device will be located). The Cordella sensor was placed in the RPA at the downturn and CardioMEMS in the LPA. The link distance for the RPA was recorded as the distance from the sensor to the reader that is on the anterior chest surface. The link distance of the LPA was measured as the distance from the sensor to the closest point on the posterior surface of the back of the patient where they will be placed (Fig. 5A, C). The RPA downturn is defined as the location in the RPA downstream of the apical bifurcation where the interlobar artery typically turns downward and posterior before further branching into a series of basal arteries feeding the lower lung lobes. The angle of this downturn was measured for analysis (Fig. 6A).

### Statistical analysis

Statistical analysis was performed using Microsoft Excel (Microsoft Inc., Redmond, WA, USA) to determine the normal distribution of the data. The distribution of data was described using the mean and standard deviation. The coefficient of determination (R^2^) between the link distance and chest circumference was calculated to assess whether there was a correlation between changes in both variables. The closer the R^2^ value was to 1, the stronger the correlation between the two variables was determined to be. The coefficient of variation (CV) was also calculated to help compare metrics of different nature with different units as it is a statistical measure of the relative dispersion of data points in a data series around a mean.

## Results

Thirty-eight heart failure patients at the Sheffield Teaching Hospitals NHS Trust were analysed, and 34 CT scan images were chosen for 3D reconstruction based on the quality of images and the measurability of pre-selected distal segments, as described in Fig. 2. 2-D transthoracic echocardiography was performed to measure the left ventricular ejection fraction. Patients were 53% male and had a mean age of 74.8 ± 12.98 years with a mean ejection fraction of 47.1 ± 12.81%. Patients were predominantly NYHA class III and IV (47.2%), 29.4% had COPD, 41.4% had hypertension, and 32.2% had atrial fibrillation (Table 1). The mean distance from the bifurcation of the main PA to the target sensor location was 4.55 ± 0.64 cm in the RPA and 7.4 ± 1.3 cm in the LPA, as seen in Fig. 4B. For implantation, pressure monitors are mounted on the delivery catheter with the nitinol anchors arranged in a compressed formation to facilitate smooth delivery through the vascular system to the point of delivery. Once at the point of deployment, the release wire is withdrawn and the nitinol anchors that support and stabilise pressure monitors within the pulmonary artery return to their preformed size and shape. The mean dimensions of both the left and right pulmonary arteries proximal to distal their course: RPA proximal (25.75 ± 4.65 mm), RPA sensor (17.15 ± 2.87 mm), and RPA distal (12.79 ± 2.92 mm); LPA proximal (26.00 ± 4.05 mm), LPA sensor (11.83 ± 2.30 mm), and LPA distal (7.39 ± 1.55 mm) (Fig. 3B). The proximal dimensions of the left–right pulmonary artery are well-matched. However, the left pulmonary artery is narrower at the point of implantation highlighting the importance of fluoroscopic imaging for vessel sizing.

**Table 1:** Demographic characteristics, comorbidities, and NYHA classification of 34 patients involved in this research project.

Patient characteristics	Analysed cohort (n)
Implanted population (n)	34
Demographics	
Age (years), mean, range ± SD	74.8 (29.0–92.0) ± 12.98
Male, % (n)	53.0 (18)
Female, % (n)	47.1 (16)
Asian race, % (n)	2.9 (1)
Caucasian race, % (n)	97.1 (33)
Comorbidities	
Myocardial infarction, % (n)	8.8 (3)
Diabetes mellitus, % (n)	23.5 (8)
Coronary artery disease, % (n)	44.1 (15)
Hypertension, % (n)	41.1 (14)
Atrial flutter/fibrillation, % (n)	32.2 (11)
COPD, % (n)	29.4 (10)
Ejection fraction mean (range) ± SD	47.1 (24.0–66.0) ± 12.81
NYHA classification	
NYHA classification I, % (n)	20.5 (7)
NYHA classification II, % (n)	32.3 (11)
NYHA classification III, % (n)	41.4 (14)
NYHA classification IV, % (n)	5.8 (2)

Once implanted the pressure monitor is read using an externally powered radiofrequency communication system placed on the back in the supine position (CardioMEMS) or the front (Cordella) in the seated position. The signal is affected by the composition of body tissue between the reader and the pressure sensor and by the distance that separates the 2 components (link distance). The mean link distance for the Cordella sensor in the right pulmonary artery to the reader on the anterior chest was 12.54 ± 1.37 mm. The average link distance for the CardioMEMS sensor in the left pulmonary artery to the reader on the posterior chest was 9.40 ± 1.43 mm (Fig. 5B). Link distance correlated with chest circumference for both RPA and LPA (R^2^ = 0.99 for both RPA and LPA, Fig. 5D). The mean downturn angle of the RPA (Cordella sensor) measured 135.5 ± 8.2 degrees across all patients. Given that the Cordella sensor is a landmark-based design, we sought to examine the coefficient of variation in sensor location (angle vs. diameter). As shown in Fig. 6B, the CV for the RPA downturn angle is 5% whereas the CV for the RPA diameter and LPA diameter are 17 and 19%, respectively meaning the angle of the downturn is more consistent across this patient population than vessel diameter at the level of LPA Zone 2.

## Discussion

Heart failure remains a cause of significant morbidity and mortality and places a large burden on healthcare expenditure^12^. With the development of PAP sensors, heart failure patient monitoring and guideline-directed medical therapy (GDMT) interventions are possible in real-time, bridging the gap between clinic visits, and allowing for proactive remote monitoring to reduce HF-related readmissions and mortality^17^.

Both the CardioMEMS and Cordella devices use micro-electromechanical systems (MEMS) and radiofrequency technology to accurately transduce pressure and communicate measurements to their respective readers. MEMS can act as sensors, receiving information from their environment and providing an electrical output signal for an external reader. The strength of the communication signal is altered by the alignment and length of the path between the sensor and the reader. Key design features influence implant location with implications for device use and accurate, accessible home readings. The CardioMEMS sensor resides in the lumen of the LPA and PAP is captured by the patient while lying supine on the reader pillow. The Cordella sensor resides against the anterior wall of the RPA and PAP is captured using a hand-held reader from either the seated or supine position. The proximal anchor of the Cordella sensor uses the principle of outward radial force to interact with and fix to the vessel wall. The pre-selected target implant location in the RPA should measure 12–26 mm to allow for proper proximal anchor engagement^18^. The vessel diameter at the site of the sensor deployment was 17.15 ± 2.87 mm in this study, well within the range of the design specifications. The distal anchor has a parent shape-set configuration approximately orthogonal to the sensor body. When deployed, the distal anchor deflects distally and, due to the shape memory effect of nitinol, exerts a bending force in the direction of its parent shape-set, towards the vessel wall, securing the sensor in place. The angle of the downturn of the RPA was 135.5 ± 8.2 degrees in this study indicating that there is adequate strain put on the anchor across this patient population to exert a sufficient bending force to secure the anchor in place and control rotation. Fixing the sensor at a known landmark, the downturn of the RPA has potential advantages including orientation and stabilization of the sensor and standardization of implanting^14,15^. Both anchors of the CardioMEMS sensor use the principle of outward radial force to interact and fix to the vessel wall. The pre-selected target vessel is within the lower lobe of either lung with the vessel directed towards the feet and back, the vessel diameter is ≥ 7 mm and has < 30-degree angulation where the body of the sensor will be placed, and the vessel diameter is 5–8 mm where the distal anchor will be placed^19^. The vessel diameter at the site of sensor body deployment was 11.83 ± 2.30 mm, well within the range of the implant recommendations. The RPA sensor location provides a sensor-to-anterior chest link distance of 12.54 ± 1.37 mm which permits signal transmission detection from the anterior chest with a hand-held reader in contrast to supine, posterior readings with the LPA implant sensors. Furthermore, the route of implantation is feasible from the groin or internal jugular vein (IJV) with either device. Jugular implantation of CardioMEMS has been demonstrated to be safe and feasible^20^. Additionally, the opportunity to take standing and ambulatory readings with the hand-held reader opens the possibility to assess patient haemodynamics while ambulating, in the clinic or the home environment.

## Conclusion

In this paper CT images of the population with sensor implants and their relationship between vessel measurements were examined. The left and right pulmonary arteries were characterized as the locations for the CardioMEMS and Cordella PA sensors. The CardioMEMS sensor has a “diameter-based” design and is typically implanted in the LPA where anatomy permits. The Cordella sensor has a “landmark-based” design meant to be deployed in the RPA, distal to the apical bifurcation, where the interlobar artery typically turns downward and posterior. Across the study population, chest circumference correlated with sensor-reader link distance with the Cordella sensor having a smaller link distance with respect to the anterior chest allowing for front-sided readings with a hand-held reader. The CardioMEMS sensor has a smaller link distance to the patient’s back, consistent with the design of the reader pillow. Analysis of pulmonary vascular anatomy with respect to PA diameter, angulation of vessels, and depth of sensor location prior to PAP sensor implantation may contribute to device choice and inform implant strategy.

### Supplementary Information

Supplementary Information.

## Data Availability

The data has been obtained from the ASPIRE Registry (Assessing the Severity of Pulmonary Hypertension in a Pulmonary Hypertension REferral Centre: research ethics number 16/YH/0352). This is a clinical registry of data collected through clinical practice. The research ethics approved process means that following a positive outcome from a review of the proposal by an independent committee database data is provided for research purposes. All data is collected from clinical practice, all patients have the opportunity to opt out of the database, and any researcher can apply for access to the data. The datasets used and/or analysed during the current study are available from the corresponding author upon reasonable request.

## Abbreviations

PAP: Pulmonary artery pressure
CHF: Congestive heart failure
RHF: Right heart failure
LV: Left ventricle
HFpEF: Heart failure with preserved ejection fraction
CHFS: Cordella heart failure system
GDMT: Guideline directed medical therapy
CT: Computerized tomography
MDCT: Multidetector computerized tomography
MEMS: Micro-electromechanical systems
HRCT: High-resolution computerized tomography
3D: 3-Dimensional
DICOM: Digital imaging and communications in medicine
PA: Pulmonary artery
RPA: Right pulmonary artery
LPA: Left pulmonary artery
CFD: Computational fluid dynamics
HF: Heart failure
IJV: Internal jugular vein
NYHA: New York Heart Association
RHC: Right heart catheterization
TA: Truncus arteriosus
