# Supplementary material for: Anatomical characterization of pulmonary artery and implications to pulmonary artery pressure monitor implantation
Source: Sci Rep. 2023 Nov 22;13:20528. doi: 10.1038/s41598-023-47612-9 (PMC10665414; doi:10.1038/s41598-023-47612-9)

Anatomical Characterization of Pulmonary Artery and Implications to Pulmonary Artery Pressure Monitor Implantation

Type of Article: Research Article

Hamza Zafar^1^, Dharshan Neelam-Naganathan^1^, Jennifer T Middleston^1^, Sarah K Binmahfooz^1^, Christian Battersby^1^, Dominic Rogers^2^, Andrew J Swift^1^, Alexander MK Rothman^1^

^1.^ Department of Infection, Immunity & Cardiovascular Disease, University of Sheffield, United Kingdom

^2.^ Department of Cardiology, Sheffield Teaching Hospital NHS

Corresponding Author:

Alex Rothman, MBChB, PhD
Division of Clinical Medicine

Faculty of Medicine, Dentistry & Health

University of Sheffield

Medical School

Beech Hill Road

Sheffield S10 2RX

Tel: +44 (0) 114 215 9500

E-Mail: a.rothman@sheffield.ac.uk

# Supplementary online document

**Pulmonary Artery Characterization Measurements for All Patients**

**Supplementary Table S1.** Raw detailed measurement data for all patients in the study. (1) is labelled for the measurement made at the most proximal site on the artery right after bifurcation. (2) is the measurement made at the site where the implant is placed. (3) is the measurement made at a site distal to (2).

| Patient ID | | Chest Circumference | RPA (1) | RPA (2) | RPA (3) | RPA LD | RPA (2) to MPA (cm) | RPA Downturn | LPA (1) | LPA (2) | LPA (3) | LPA LD | LPA (2) to MPA |
| --- | --- | --- | --- | --- | --- | --- | --- | --- | --- | --- | --- | --- | --- |
| 1 |  | |  |  |  |  |  |  |  |  |  |  |  |
| 2 | 104 | | 24 | 17 | 17 | 14 | 4 | 125 | 26 | 13 | 7 | 11 | 7 |
| 3 | 113 | | 39 | 25 | 17 | 12 | 6 | 141 | 37 | 13 | 7 | 10 | 9 |
| 4 | 89 | | 25 | 18 | 12 | 11 | 4 | 149 | 25 | 17 | 11 | 9 | 6 |
| 5 | 111 | | 26 | 20 | 14 | 14 | 5 | 130 | 30 | 13 | 7 | 11 | 8 |
| 6 | 105 | | 30 | 19 | 16 | 13 | 5 | 152 | 25 | 11 | 9 | 11 | 9 |
| 7 | 96 | | 26 | 16 | 15 | 10 | 5 | 152 | 21 | 12 | n/a | 7 | 5 |
| 8 | 78 | | 26 | 20 | 13 | 10 | 4 | 145 | 25 | 12 | 9 | 8 | 8 |
| 9 | 100 | | 26 | 16 | 10 | 14 | 4 | 130 | 26 | 9 | 6 | 8 | 6 |
| 10 | 105 | | 39 | 26 | 18 | 15 | 4 | 137 | 36 | 14 | 11 | 10 | 8 |
| 11 |  | |  |  |  |  |  |  |  |  |  |  |  |
| 12 | 106 | | 30 | 17 | 14 | 13 | 6 | 123 | 26 | 13 | 6 | 10 | 9 |
| 13 | 89 | | 31 | 16 | 14 | 12 | 5 | 136 | 29 | 13 | n/a | 9 | 5 |
| 14 | 95 | | 22 | 15 | 11 | 13 | 5 | 136 | 22 | 9 | 5 | 10 | 8 |
| 15 | 107 | | 28 | 16 | 12 | 15 | 4 | 143 | 30 | 12 | 7 | 10 | 8 |
| 16 | 103 | | 26 | 16 | 14 | 13 | 4 | 125 | 27 | 10 | 7 | 9 | 6 |
| 17 | 110 | | 29 | 18 | 14 | 14 | 5 | 129 | 31 | 15 | 9 | 11 | 7 |
| 18 | 97 | | 23 | 15 | 12 | 12 | 6 | 132 | 21 | 12 | 8 | 10 | 8 |
| 19i | 104 | | 25 | 20 | 17 | 13 | 5 | 145 | 23 | 15 | n/a | 11 |  |
| 20 | 104 | | 24 | 18 | 10 | 12 | 4 | 143 | 27 | 14 | 8 | 11 | 10 |
| 21 | 120 | | 24 | 17 | 10 | 13 | 4 | 140 | 27 | 11 | 8 | 11 | 7 |
| 22 | 107 | | 21 | 15 | 10 | 13 | 4 | 127 | 23 | 10 | 7 | 11 | 8 |
| 23 | 102 | | 27 | 20 | 18 | 14 | 5 | 144 | 30 | 10 | 7 | 9 | 9 |
| 24 | 98 | | 25 | 20 | 14 | 15 | 4 | 129 | 28 | 16 | 9 | 9 | 8 |
| 25 | 107 | | 23 | 15 | 10 | 13 | 4 | 128 | 23 | 12 | 7 | 11 | 5 |
| 26 | 104 | | 29 | 15 | 11 | 13 | 5 | 138 | 26 | 8 | 5 | 11 | 6 |
| 27 | 103 | | 23 | 17 | 16 | 11 | 4 | 135 | 27 | 9 | 6 | 10 | 8 |
| 28 | 101 | | 25 | 16 | 13 | 12 | 4 | 124 | 26 | 11 | 6 | 8 | 6 |
| 29 | 83 | | 22 | 12 | 7 | 10 | 5 | 138 | 24 | 10 | 7 | 8 | 6 |
| 30 | 81 | | 21 | 16 | 11 | 12 | 4 | 144 | 26 | 14 | 8 | 6 | 8 |
| 31 | 102 | | 26 | 16 | 16 | 11 | 5 | 127 | 25 | 12 | 9 | 9 | 9 |
| 32 |  | |  |  |  |  |  |  |  |  |  |  |  |
| 33 |  | |  |  |  |  |  |  |  |  |  |  |  |
| 34 | 87 | | 28 | 16 | 10 | 12 | 5 | 129 | 30 | 11 | 7 | 8 | 8 |
| 35 | 95 | | 18 | 15 | 11 | 12 | 5 | 142 | 20 | 7 | 5 | 7 | 7 |
| 36 | 103 | | 20 | 14 | 9 | 13 | 4 | 128 | 19 | 10 | 6 | 10 | 7 |
| 37 | 97 | | 20 | 14 | 9 | 11 | 5 | 134 | 20 | 10 | 6 | 7 | 7 |
| 38 | 76 | | 23 | 17 | 11 | 13 | 4 | 128 | 24 | 11 | 6 | 7 | 8 |

**Supplementary Table S2.** Raw detailed descriptive statistical analysis for all patients in the study. (1) is labelled for the measurement made at the most proximal site on the artery right after bifurcation. (2) is the measurement made at the site where the implant is placed. (3) is the measurement made at a site distal to (2).

| Patient ID | Chest Circumference | RPA (1) | RPA (2) | RPA (3) | RPA LD | RPA (2) to MPA (cm) | RPA Downturn | LPA (1) | LPA (2) | LPA (3) | LPA LD | LPA (2) to MPA |
| --- | --- | --- | --- | --- | --- | --- | --- | --- | --- | --- | --- | --- |
| Average | 99.47 | 25.75 | 17.15 | 12.79 | 12.54 | 4.55 | 135.52 | 26.00 | 11.83 | 7.39 | 9.40 | 7.43 |
| STD | 10.06 | 4.65 | 2.87 | 2.92 | 1.37 | 0.64 | 8.24 | 4.05 | 2.30 | 1.55 | 1.43 | 1.26 |
| Min | 76.27 | 17.75 | 12.00 | 6.60 | 10.00 | 3.53 | 123.34 | 18.88 | 7.09 | 4.82 | 6.49 | 4.96 |
| Q1 | 95.26 | 23.05 | 15.25 | 10.32 | 11.73 | 4.07 | 128.55 | 23.33 | 10.22 | 6.44 | 8.24 | 6.43 |
| Median | 102.57 | 25.29 | 16.00 | 12.58 | 12.65 | 4.45 | 135.29 | 25.60 | 11.65 | 7.25 | 9.66 | 7.66 |
| Q3 | 105.00 | 27.86 | 18.25 | 14.89 | 13.43 | 5.00 | 143.18 | 28.36 | 13.37 | 8.38 | 10.71 | 8.31 |
| Max | 120.41 | 39.25 | 26.00 | 17.83 | 14.90 | 5.78 | 152.32 | 36.67 | 17.49 | 11.30 | 11.33 | 9.60 |
| Box1 | 95.26 | 23.05 | 15.25 | 10.32 | 11.73 | 4.07 | 128.55 | 23.33 | 10.22 | 6.44 | 8.24 | 6.43 |
| Box2 | 7.31 | 2.24 | 0.75 | 2.26 | 0.93 | 0.37 | 6.74 | 2.27 | 1.43 | 0.82 | 1.42 | 1.23 |
| Box3 | 2.43 | 2.57 | 2.25 | 2.32 | 0.78 | 0.56 | 7.89 | 2.77 | 1.72 | 1.13 | 1.05 | 0.65 |
|  |  |  |  |  |  |  |  |  |  |  |  |  |
| Top | 15.41 | 11.40 | 7.75 | 2.94 | 1.48 | 0.78 | 9.14 | 8.31 | 4.13 | 2.92 | 0.63 | 1.29 |
| Bottom | 18.99 | 5.30 | 3.25 | 3.72 | 1.73 | 0.55 | 5.21 | 4.45 | 3.13 | 1.62 | 1.75 | 1.47 |
| Coefficient of variation | 10% | 18% | 17% | 23% | 11% | 14% | 6% | 16% | 19% | 21% | 15% | 17% |
| Quartile coefficient of dispersion |  | 9% | 9% | 18% | 7% | 10% | 5% | 10% | 13% | 13% | 13% | 13% |

**Representative CT Pulmonary Angiogram Windows for Measurement**

**Supplementary Figure S1.** Right Pulmonary Artery Segment Length from implant to MPA. By using the 3 planes and the 3D reconstruction, the slice that went right in the middle of the vessel from the downturn to the bend in the RPA was found (Figure 1a). After doing so, using the axial view, the distance from the RPA downturn to the bend of the artery was first measured. Then using the 3 views, we once again found a slice that cut right across from the RPA bend to the MPA (Figure 1b). Using the axial plane, the length was measured from the centre of the bend to the MPA.

1a.


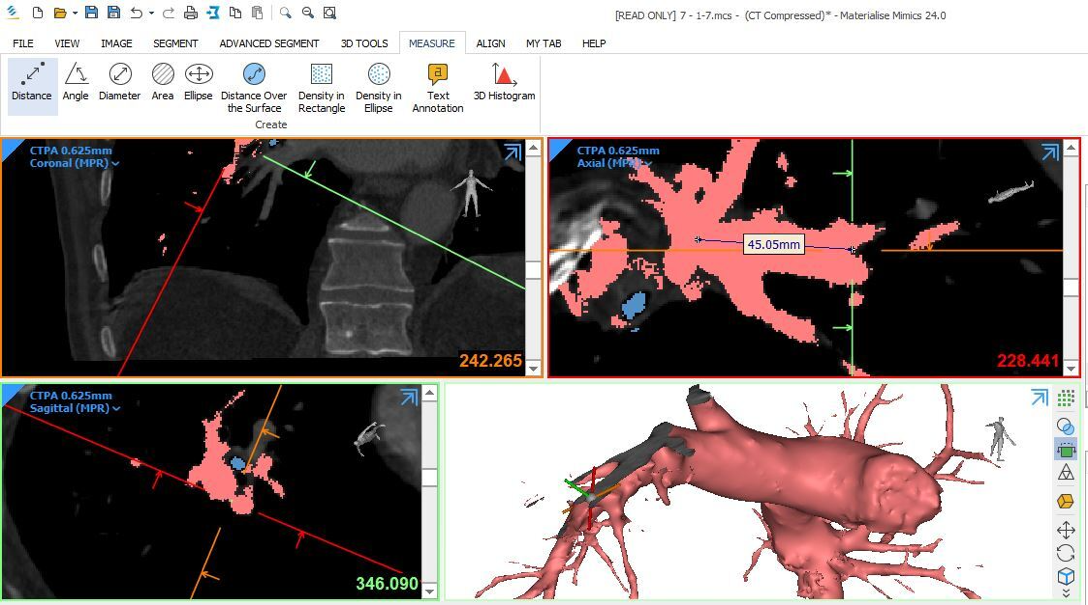


1b.


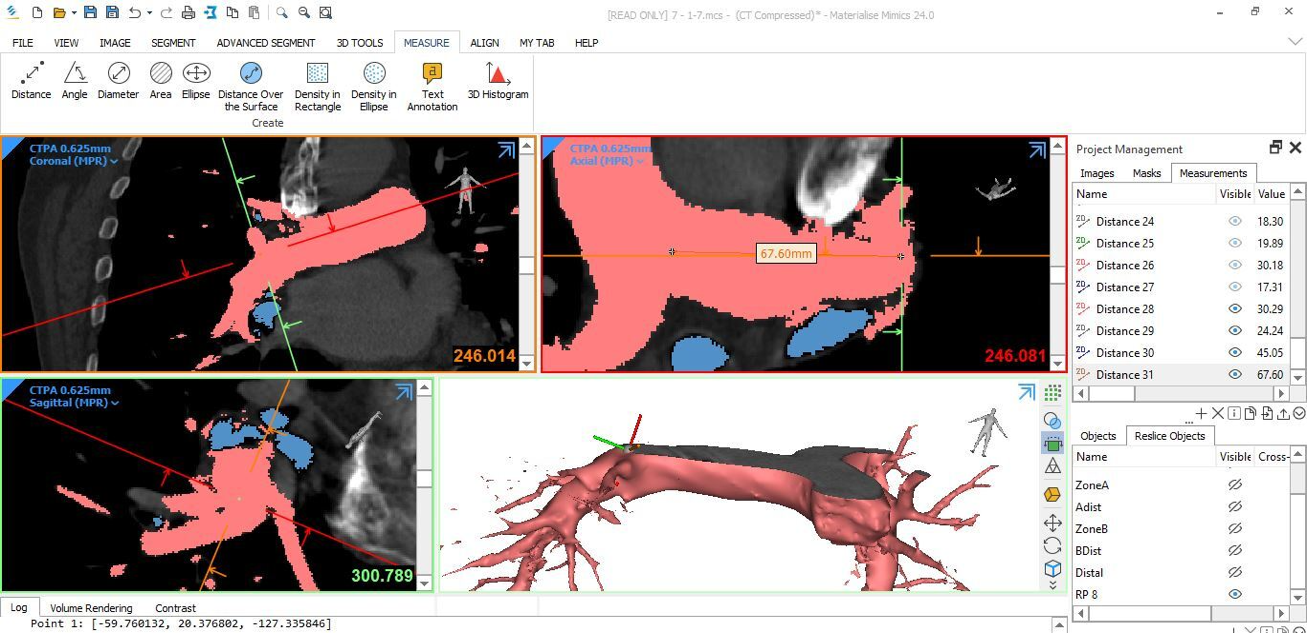


**Supplementary Figure S2.** Right Pulmonary Artery Downturn Angle. This is the site where the Cordella sensor would be placed. The angle was measured using the axial slice after determining that the measurement was being made at the centre of the vessel using the coronal and sagittal view and the reconstructed 3D model.


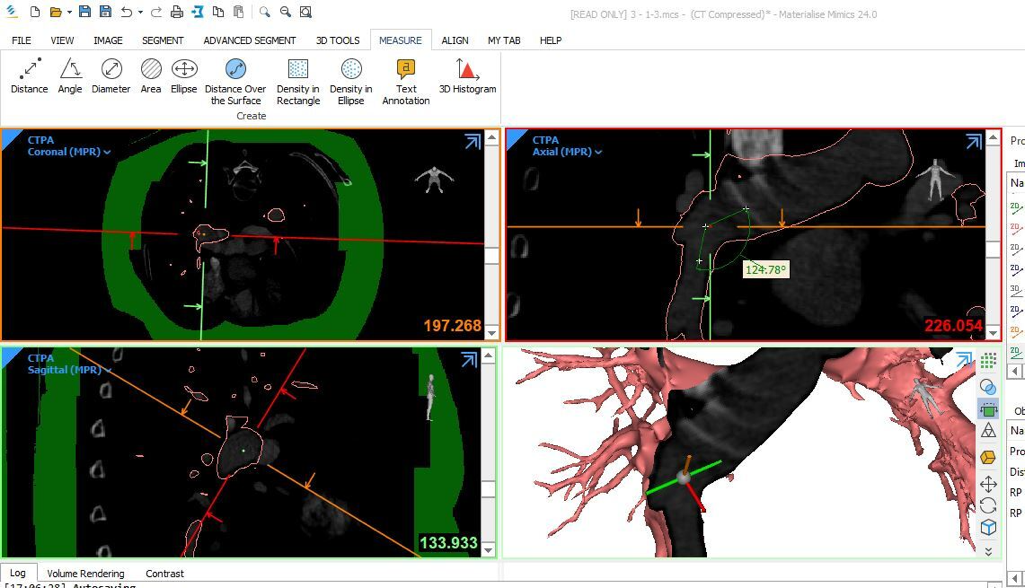

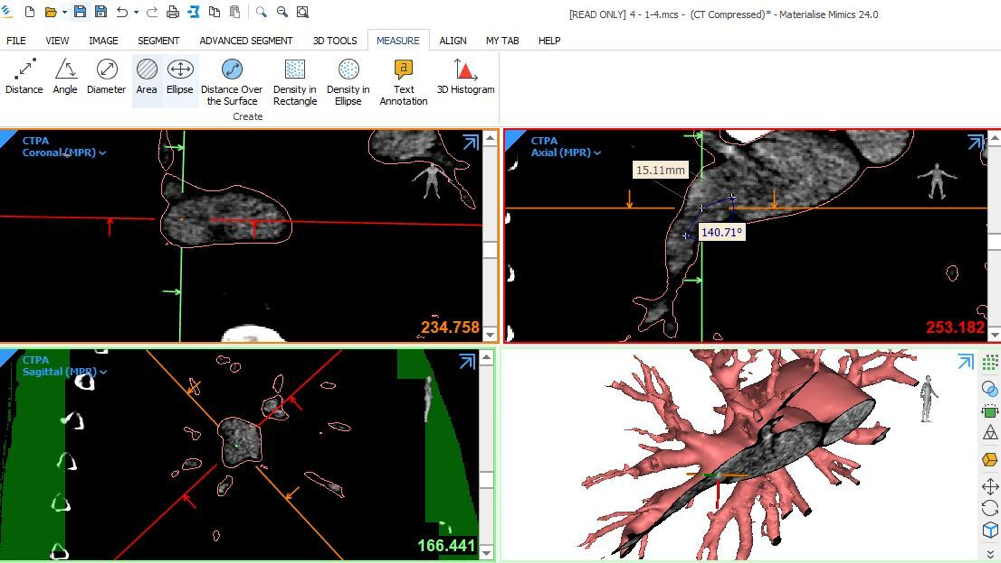


**Supplementary Figure S3.** Left Pulmonary Artery Segment Length from implant location to MPA. By using the 3 planes and the 3d reconstruction, the slice that went right in the middle of the vessel from the location of the CardioMEMS implant in the LPA to the proximal bend of the LPA was found (Figure 3a). After doing so, using the axial view, the distance from the implant point to the bend of the artery was first measured. Then using the 3 views, a slice that cut right across from the LPA bend to the MPA was found (Figure 3b). Using the axial plane, the length was measured from the centre of the bend to the MPA.

3a.


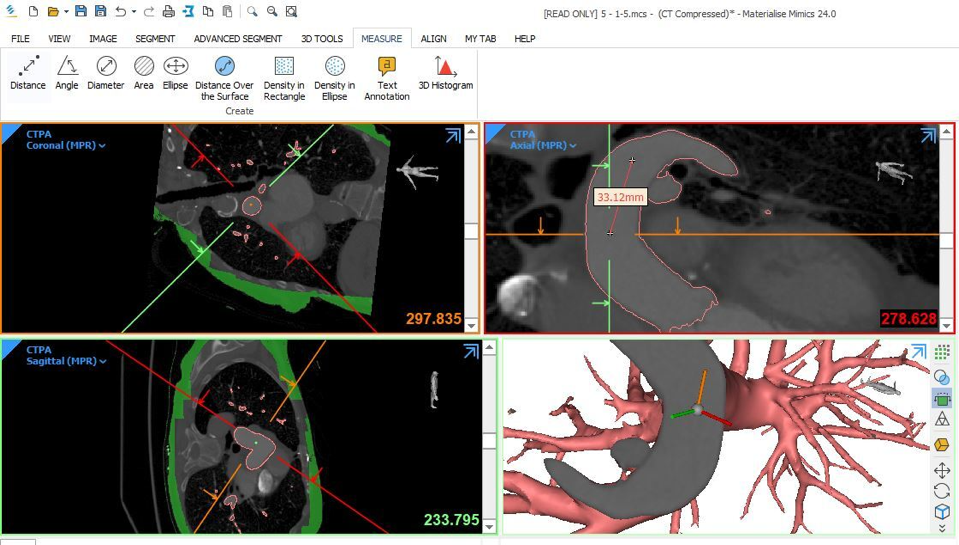


3b.


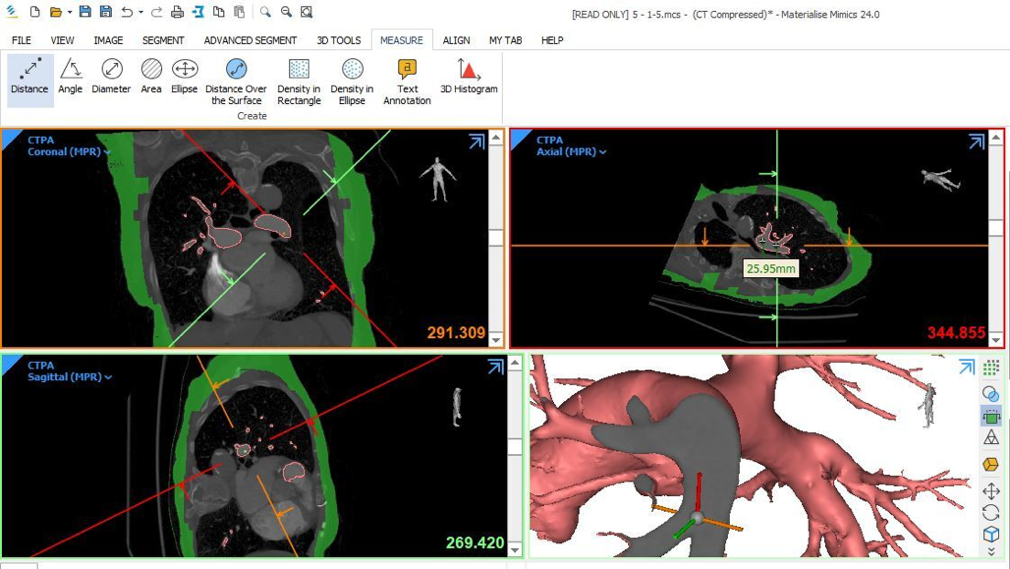


**Supplementary Figure S4.** Left Pulmonary Artery Link Distance. The posterior skin and muscle of the back of the patient are illustrated by the green shade. The link distance was measured by measuring the shortest distance from the point of implantation on the centre of the vessel to the point on the distal border of the back using the axial slice.


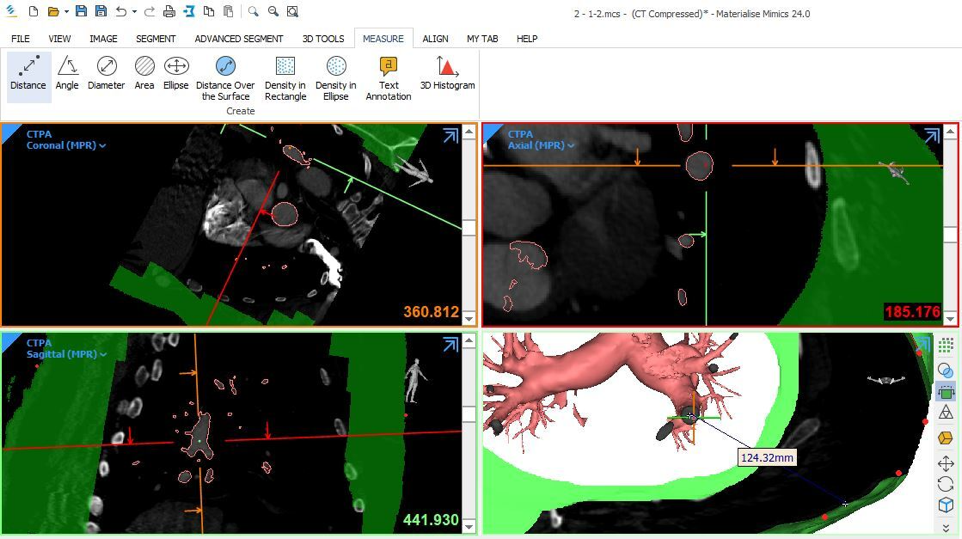

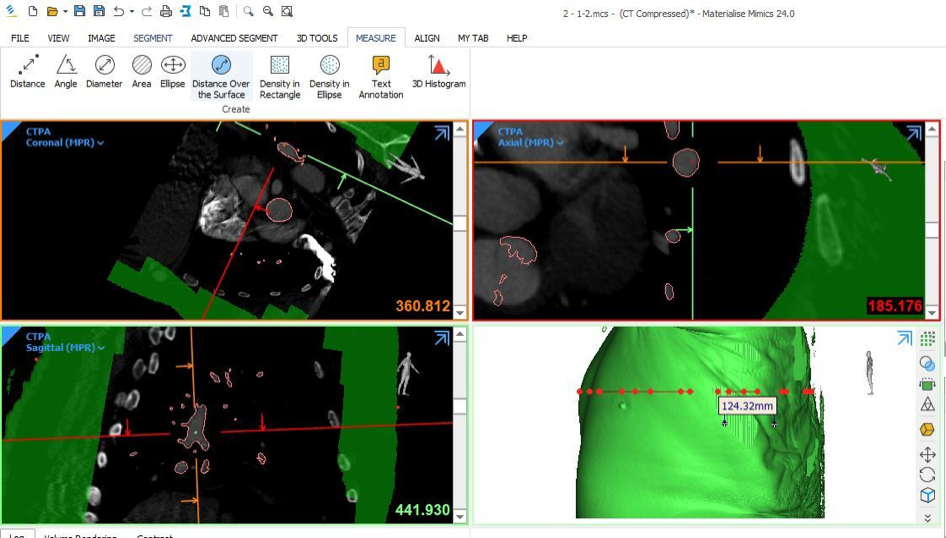


**Supplementary Figure S5.** Left Pulmonary Artery Diameter. The diameter was measured in the axial plane for each vessel as shown in the top right window. The coronal and sagittal planes and the 3D reconstruction were used to ensure that the measurement was made in the centre of the vessel on a slice that followed the path of the vessel rather than cutting it in an irregular pattern. If the slice did not follow the path of the vessel, then it could distort the measured vessel diameter. Each slice and measurement were confirmed to be accurate by ensuring that the axes (the green, red and yellow axes on the sagittal and coronal views) followed the direction of the vessel at the point being measured.


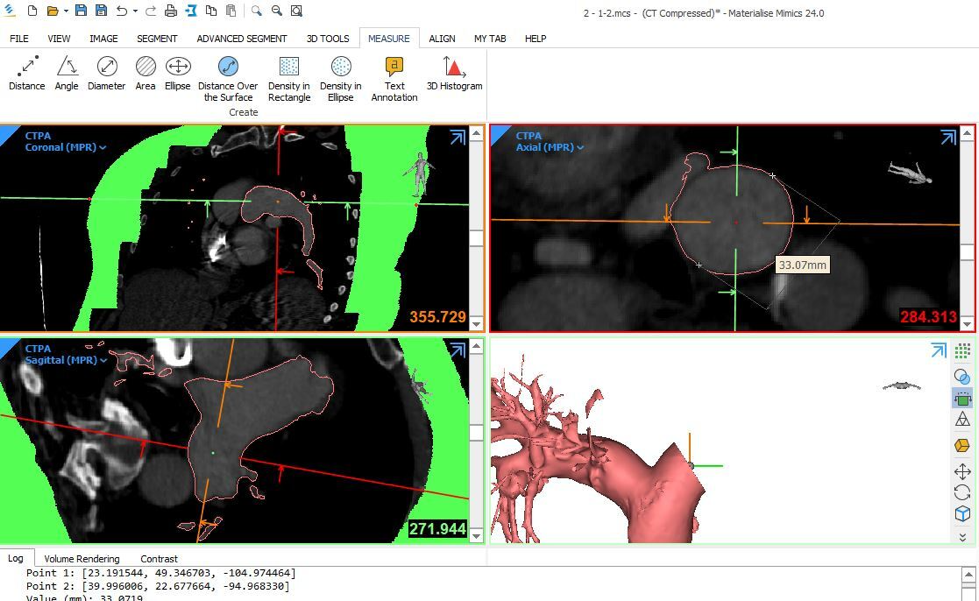

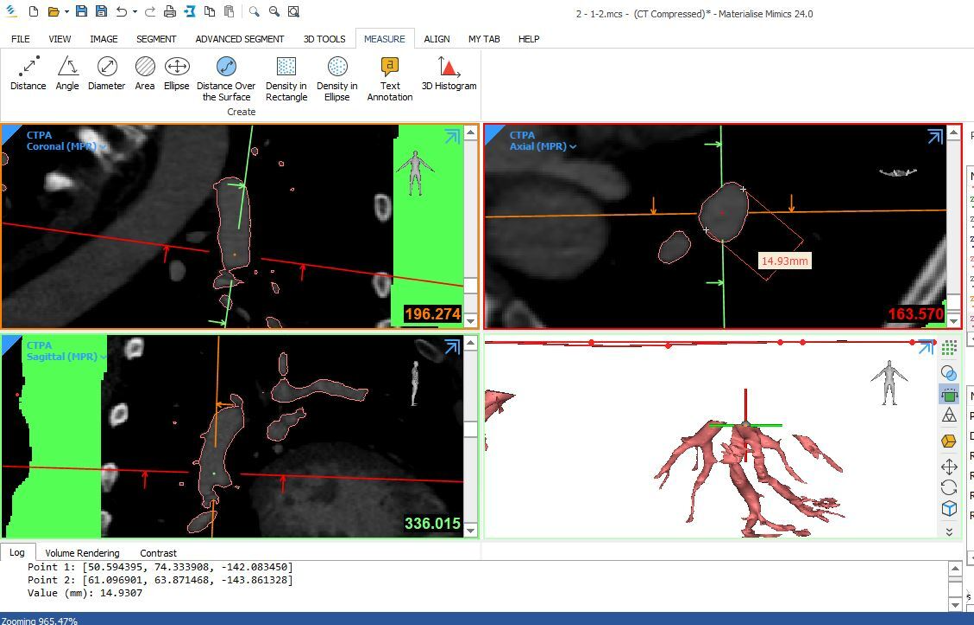

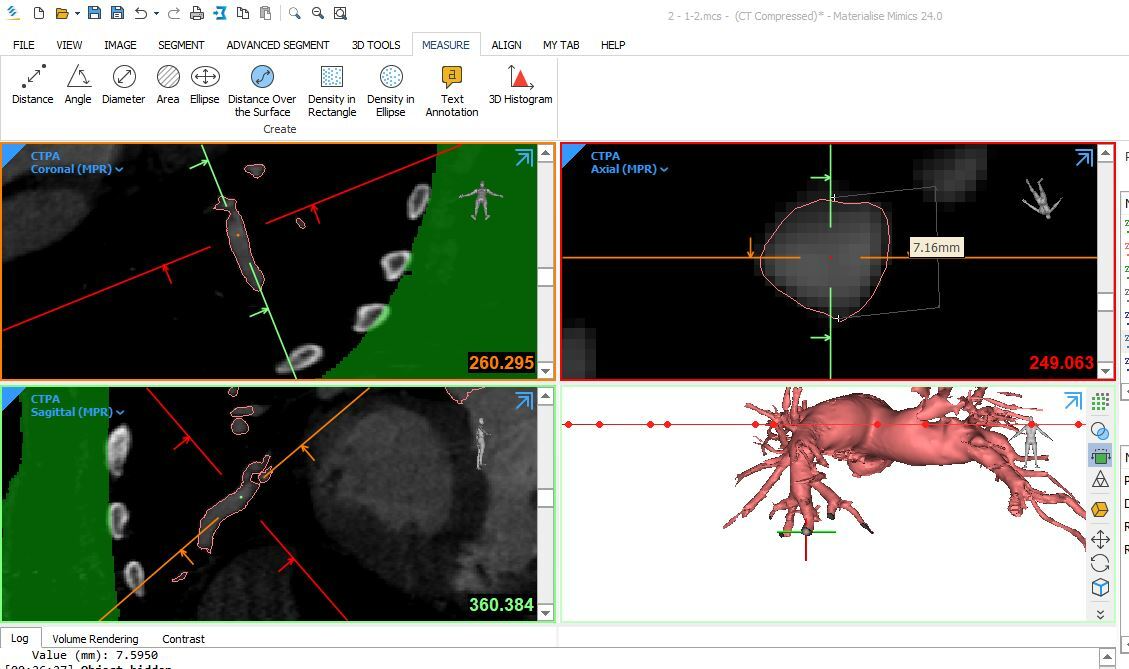

Supplement: Supplementary file 1 — Supplementary Information. [file 41598_2023_47612_MOESM1_ESM.docx]
